# Health system evaluation: new options, opportunities and limits

**DOI:** 10.2471/BLT.23.289712

**Published:** 2024-05-28

**Authors:** Kevin Croke, Edwine Barasa, Margaret E Kruk

**Affiliations:** a Harvard TH Chan School of Public Health, 677 Huntington Ave, Boston, MA 02459, United States of America.; b Kenya Medical Research Institute/Wellcome Trust Research Programme, Nairobi, Kenya.

High-quality evaluation is critical for health systems because it enables the best use of scarce resources and helps policy-makers learn what works best in their setting. What works cannot be assumed: since health systems are complex, system reforms have long causal chains and multiple interacting components. Many promising health system interventions, even those that increase intervention coverage, fail to improve health outcomes such as mortality and morbidity. However, many health system reforms, especially in low- and middle-income countries, are never evaluated due to data limitations and scarce resources for health systems research.

Counterfactual-based designs (that is, evaluations in which research design enables inference about the causal impact of a policy) are challenging to implement for health system reforms. Reforms are often applied to the whole system, leaving no obvious control group, or are assigned to high-level administrative units, limiting the number of treated and comparison units. Reforms may be targeted to areas or groups for political reasons, limiting ability to randomize and constraining generalizability. Many researchers have seen these challenges as reasons why rigorous evaluations of complex health system reforms are unlikely to succeed, promoting instead realist designs rooted in largely qualitative methods. While these methods have important strengths, recent innovations in evaluation methods, approach and data have also opened new possibilities for health system evaluation. These innovations include large-scale randomized health system trials, causal inference methods for better non-randomized inference and new technologies for data collection and analysis, including big data. These applications, which have emerged from disparate academic disciplines and from practice, may not be fully appreciated by applied health systems researchers.

A first innovation is the growing application of randomized controlled trials to system questions. Randomization has often been considered infeasible for health system questions. For example, a study shows that 79% (139/176) of intervention evaluation papers in top medical, economics, and health services journals in the United States of America were randomized controlled trials, compared to fewer than one fifth of health delivery (that is, health system) papers.^1^ However, use of randomized designs for health system evaluation in low- and middle-income countries is increasing. Recent examples include evaluations of performance-based financing in Nigeria;^2^ subsidized health insurance in Indonesia;^3^ community health worker recruitment and supply chain organization for medicine delivery in Zambia;^4^^,^^5^ and point-of-care quality interventions in northern India.^6^ Beyond maximizing internal validity, randomized controlled trials also allow researchers to test causal mechanisms, including predictions derived from theory. Direct tests of theory can enable systematic, linked accumulation of knowledge on important questions. These studies have had important implications for practice, for example by limiting enthusiasm about the potential of performance-based financing or coaching interventions to improve quality of care. In the absence of high-quality randomized evidence, advocates on opposing sides might have continued to cite competing non-randomized studies. Randomization can be difficult for political and practical reasons. Yet these proofs by existence demonstrate that large-scale, system-level randomized controlled trials should not be considered impossible beforehand, particularly when governments and/or sponsors are keen to learn and engage early in the process.

When randomization is not possible, rigorous evaluations of health system reforms with careful attention to counterfactual comparison has become increasingly feasible using methods such as difference-in-difference or regression discontinuity designs. Application of these methods has been hindered in the past by limited data availability. Yet the data picture has changed for the better in many low- and middle-income countries. Household surveys such as the Demographic and Health Surveys have expanded their topical and geographic coverage, and are now routinely geocoded. When multiple national survey rounds take place before and after programme scale-up, difference-in-difference research designs can often be used to estimate the impact of policy. Administrative data from vital registration systems, national health management information systems or national insurance programme claims data, long used in high-income countries, are increasingly usable for such research in middle-income countries. Brazil provides several examples: one study uses the staggered expansion of Brazil’s *Programa Saúde da Família* in a difference-in-difference framework to demonstrate that the programme substantially reduced infant and maternal mortality.^7^ By contrast, and using similar data and methods, another study shows that the *Mais Medicos* programme, in which expatriate doctors were deployed to underserved communities in Brazil, did not affect infant mortality.^8^

This approach has been more limited in regions that lack comprehensive vital registration. In these settings, administrative data-based evaluations are often limited to utilization data, aggregated on the platform of the health management information system DHIS2. These data systems have faced challenges with data quality as well as completeness. Health management information system data capture what happens in facilities, missing outcomes (including mortality) that occur at home, and typically do not capture individual-level data. Yet even with these limitations, these data have been increasingly leveraged in studies for which service utilization is the primary outcome. For example, these data have been used to demonstrate the impact of the coronavirus disease 2019 (COVID-19) pandemic on health system utilization.^9^

These challenges of data completeness and quality have generated enthusiasm about potential uses of technology, including big data, to enable more health system evaluation, including in settings with limited administrative data. Experience so far demonstrates promise but also grounds for caution. On the positive side, digital technologies have been used to improve surveys, digitize routine data collection, expand demographic surveillance systems and integrate remotely sensed data. Mass mobile phone ownership has opened new possibilities for mobile data collection in low- and middle-income countries: during COVID-19, many researchers and institutions successfully implemented mobile phone data collection protocols. For example, recent multicountry mobile phone surveys have effectively captured health system performance data in low-income countries.^10^ Digital technologies have also enabled expansion of health and demographic sentinel sites to nationally representative scale in some settings, such as Mozambique’s countrywide mortality surveillance for action system. New digital platforms, such as the socioeconomic high-resolution rural-urban geographic platform for India,^11^ can now aggregate surveys, geospatial data and geographically coded administrative data. 

Larger scale applications of big data (beyond survey and administrative data) have been creatively leveraged for some forms of health research in low- and middle-income countries. Mobile phone call data records have been used to study population movement, informing disease transmission dynamics. Social media posts have been used to predict disease outbreaks. Researchers have envisioned a future in which passively collected health status measures from wearable devices, or health utilization from facility-based sensors, via the Internet of Things, can be used for evaluation. In development economics, researchers increasingly benefit from the fact that variables of interest such as night-time luminosity, housing infrastructure, temperature, pollution or land use are now observed at high frequency and resolution by satellites or other remote sensing apparatuses. Researchers train machine learning algorithms to measure poverty based on these observations, opening scope for new forms of economic and social policy evaluation.

Yet application of these data sources to health system policy evaluations in low- and middle-income countries is still nascent. Many public health applications of big data are used for mapping or prediction, which is extremely useful but distinct from policy evaluation. Key health outcomes of interest such as service utilization, health status, financial risk protection or population attitudes cannot be measured by satellites or other remote sensing tools. The same incomplete electrification and digitization of health facilities that currently limits evaluation designs using DHIS2 are likely to render big data from wearables and facility-based sensors unreliable for national-scale health system evaluation studies. New technologies can generate data with greater temporal and spatial coverage. However, they do not solve the health system evaluation problem because even when data is abundant, strong research designs remain critical. New data sources open promising areas for health systems evaluation, but they cannot substitute for careful research design. Effective health system inquiry requires robust theoretical frameworks, supported by improvements in underlying administrative and vital registration data systems.

New approaches must also be rooted in an appreciation of the practicalities of policy change. Evaluation strategies must be compatible with a plausible theory of how organizations (including governments) learn, and how they make policy decisions.^12^ Health policy rarely changes based only on evidence. Policy evolves over time as competing coalitions of experts, politicians and stakeholders push for their preferred solutions. Evidence is only one input into this mix, along with values, public opinion, interest group pressure, previous studies, and ideological and intellectual predispositions of policy-makers. Policy-makers’ willingness to change their minds based on new evidence may be as much a function of the strength of their relationship and the depth of their trust with researchers, as it is with the technical rigour of the evidence.

Together with improved data and evaluation methods, investment is needed in the institutions that implement high-quality evaluations and participate in ongoing policy dialogues about the future of the health system. These institutions comprise the health research and policy community in low- and middle-income countries, including academia, think tanks, research units embedded in ministries and evidence-oriented nongovernmental organizations. These organizations are also the natural constituency to press governments to invest their own resources in better statistical systems, including both routine and survey data, which will enable better evaluation on an ongoing basis. Institutionalization of this process is not just a key ingredient in policy translation: it can also help strengthen the sustainability of evaluation and health system learning over time.
